# Socioeconomic disparities in income, education and geographic location for hypertension among Thai adults: Results from the National Socioeconomic Survey

**DOI:** 10.12688/f1000research.12709.1

**Published:** 2017-10-13

**Authors:** Atthawit Singsalasang, Wongsa Laohasiriwong, Nattapong Puttanapong, Teerasak Phajan, Suwanna Boonyaleephan

**Affiliations:** 1 Faculty of Public Health, Khon Kaen University, Khon Kaen, 40002, Thailand; 2 Faculty of Public Health and Research and Training Center for Enhancing Quality of Life for Working Age People, Khon Kaen University, Khon Kaen, 40002, Thailand; 3 Faculty of Economics, Thammasat University, Bangkok, 12121, Thailand; 4 Sirindhorn College of Public Health Khon Kaen, Khon Kaen, 40000, Thailand; 5 Faculty of Nursing, Khon Kaen University, Khon Kaen, 40002, Thailand

**Keywords:** Hypertension, socioeconomic disparity, income, education, geographic disparities, National Socioeconomic Survey, Thailand

## Abstract

**Background:** Hypertension (HT) has been one of the leading global risk factors for health and the leading cause of death in Thailand for decades. The influence of socioeconomic factors on HT has been varied and inconclusive. The aim of this study was to determine the association between socioeconomic determinants and HT in Thailand.

**Methods:** This study used data from the National Socioeconomic Survey, a cross-sectional study that was conducted by the National Statistical Office of Thailand in the years 2005, 2006 and 2007. In our analysis, data were collected on gender, age, marital status, smoking status, education, status of work, occupation, current liability (short-term debt), household monthly income, residential area, region and previously diagnosed HT by a physician.

**Results:** The odds of having HT were significantly higher among those who had household monthly income, education, residential area and region. The participants who had monthly income of <10001 baht (2005: AOR = 3.19, 95%CI:1.47 - 6.92; 2006: AOR 2.53, 95%CI:1.37 - 4.69; 2007: AOR = 3.35, 95%CI: 1.97 - 7.00), were living in Bangkok compared with the Northeast region (2005: AOR = 1.72, 95%CI:1.37 - 2.17; 2006: AOR =  2.44, 95%CI: 1.89 - 3.13; 2007: AOR =  2.63, 95%CI 2.08 - 3.45), lived as an urban resident (2005: AOR= 1.32, 95%CI: 1.12 - 1.56; 2006: AOR= 1.21, 95%CI: 1.02 - 1.43; 2007: AOR= 1.47, 95%CI: 1.18 - 1.62), and finished primary education (2005: AOR =1.21, 95%CI: 1.03 - 1.43; 2006: AOR= 1.23, 95%CI: 1.04 - 1.46; 2007: AOR= 1.18, 95%CI: 1.01 - 1.38) when controlling for other covariates.

**Conclusion:** This study indicated that socioeconomic disparity has an influence on HT. Those with low educational attainment, low income, lived in urban regions, and were metropolitan residents (Bangkok) were vulnerable to HT.

## Introduction

Hypertension (HT) is one of the top modifiable risk factors for cardiovascular diseases (CVD), a cause of morbidity and mortality worldwide ^1, 2^. In Thailand, statistics for 2003, 2008 and 2013 indicated that the morbidity rate per 100,000 population for HT were 389.80, 860.53 and 1621.72, respectively, which shows an exponential increase ^3^. In 2025, HT patients will likely to increase to 1.56 billion cases globally ^4^. Moreover, half of HT patients die from ischemic heart disease and stroke caused by HT ^3, 4^. Many studies had found that there were several factors related to the occurrence of HT. There are some known individual factors consisting of the non-modifiable factors of age ^5– 9^, gender ^5, 7– 11^, and having a family history of HT ^9, 11, 12^, and some behavioural, modifiable factors, including being overweight/obese ^6, 7, 10, 12– 14^, smoking ^11, 15^, physical inactivity ^5, 11^, high dietary salt intake ^5, 15^, alcohol consumption ^9, 12, 16, 17^, and stress ^6^. There are also factors of socioeconomic status (SES) that are correlated with HT, namely education ^6– 9, 12, 18– 20^, occupation ^6, 9, 15^, economic status ^17, 21, 22^, income ^17, 20^, and residential area ^6, 11, 15, 16^. Therefore, there are a variety of factors, both individual and SES, that have been previously associated with HT.

Previous studies on HT in Thailand ^5, 11, 17^ were inconclusive regarding whether SES has any influence on HT. Studies on the association between SES and HT have been sparse, and the results are conflicting. Therefore, a large-scale study on HT and more focused research to determine whether disparity in socioeconomic effects on health status is needed. For these reasons, the objective of this study is to examine the association between the SES and HT among the Thai adult people.

## Methods

This study used data from the National Socioeconomic Survey (NSS), conducted in 2005, 2006 and 2007 by the National Statistical Office (NSO) of Thailand. The questionnaires collected information on gender, age, marriage status, smoking status, education, occupational, status of work, household monthly income, current liabilities (short-term debt), residential area, region and previously diagnosed HT by a physician. The outcome, HT, was classified into two categories: having HT and not having HT.

### Study design and sample size

The cross-sectional survey was conducted by the NSO of Thailand. The survey used a stratified two-stage random sampling technique to select a nationally representative sample to respond to a structured questionnaire from all 76 provinces in Thailand. There were altogether 76 strata, each stratum was divided into two parts according to the type of local administration, namely, municipal areas and non-municipal areas. Selection of primary sampling, i.e. the sample selection of blocks/villages, was performed separately and independently in each part using probability proportional to the total number of households in that block or village. In the second step, the selection of secondary sampling units, i.e. private sampled households, were selected using the systematic method in each type of local administration (details of this sampling are available at http://web.nso.go.th/survey/house_seco/meth.pdf). Ultimately, there were a total of 16,306, 16,539 and 16,488 participants in 2005, 2006 and 2007, respectively, who met the inclusion criteria of Thai nationality and aged 15 years old and above were included in this analysis.

### Statistical analysis

The characteristics of the participants were described using frequency and percentage for categorical variables and the mean and standard deviation for continuous variables. Crude odds ratios (OR), adjusted odds ratios (AOR) and 95% confidence intervals (CI) were calculated using bivariate and multiple logistic regression analysis to estimate the association between independent variables with HT. To obtain AOR for the effects of independent variables on HT, variables were placed in an initial model, and those with a p-value less than 0.25 were included in multivariate modelling. Backward elimination was used as the method for variable selection to obtain the final model. All analyses were performed using Stata version 13.0 (Stata Corp, College Station, TX). The magnitudes of effects were determined using AOR and 95% CI. A p-value less than 0.05 was considered statistically significant. All statistical tests were two-sided.

### Ethical statement

The NSS study obtained signed consent forms before enrolling participants. Confidentiality of the data was fully assured. The Ethical Committee of Khon Kaen University approved the exemption for ethical approval of this study (reference no. HE 582314). The NSO administrative board approved the research team to use the data (reference no.050601/1441).

## Results

The baseline characteristics of the 16,306 participants in 2005, the 16,539 participants in 2006 and the 16,488 participants in 2007 were as follows: The majority of the participants were women (53.53%, 53.61%, 53.58%, respectively); average ages were 42.23 ± 16.99 SD, 42.56 ± 17.17 SD and 43.04± 17.39 SD years old; most of the participants had monthly household income <10,001 baht (89.47%, 89.94%, 89.57%, respectively); about a half of participants completed primary education (55.08%, 54.10%, 53.27%, respectively); the majority lived in rural areas (60.70%, 61.72% and 62.77%, respectively); the highest proportion of participants was from the Northeast region (27.45%, 27.56% and 28.03%, respectively); prevalence of smoking was 28.92%, 28.27% and 26.98%, respectively ( Table 1).

**Table 1.** Demographic and socioeconomic characteristics of participants in the National Socioeconomic Survey of Thailand for the years 2005–2007.

Characteristics	2005 (n = 16,306)		2006 (n = 16,539)		2007 (n = 16,488)	
	N	%	N	%	N	%
**Gender** Male Female	7,577 8,729	46.47 53.53	7,673 8,866	46.39 53.61	7,653 8,835	46.42 53.58
**Age (years)** <35 ≥35 - <45 ≥45 - <55 ≥55 - <65 ≥65	5,937 3,570 2,933 1,854 2,012	36.41 21.89 17.99 11.37 12.34	5,839 3,637 2,998 2,022 2,043	35.30 21.99 18.13 12.23 12.35	5,635 3,556 3,061 2,110 2,126	34.18 21.57 18.57 12.80 12.89
Mean (SD)	42.23 (16.99)		42.56 (17.17)		43.04 (17.39)	
Median (Min - Max)	41 (15 - 98)		41 (15 - 98)		42 (15 - 99)	
**Marriage status** Single Married Widowed/Separated	3,720 10,708 1,878	22.81 65.67 11.52	3,718 10,886 1,935	22.48 65.82 11.70	3,740 10,719 2,029	22.68 65.01 12.31
**Smoking status** No Yes	11,590 4,716	71.08 28.92	11,864 4,675	71.73 28.27	12,040 4,448	73.02 26.98
**Education** High (Upper primary school) Low (Primary School)	7,325 8,981	44.92 55.08	7,592 8,947	45.90 54.10	7,705 8,783	46.73 53.27
**Status of work** No Yes	4,227 12,079	25.92 74.08	4,348 12,191	26.29 73.71	4,299 12,189	26.07 73.93
**Occupation** Government officer Private business Personnel/employee Agriculture/labour	1,082 2,698 3,347 9,179	6.64 16.55 20.53 56.29	3,267 1,308 3,653 8,311	19.75 27.66 22.09 50.25	1,133 3,222 3,132 9,001	6.87 19.54 19.00 54.59
**Current liabilities (Short-term debt)** No Yes	11,461 4,845	70.29 29.71	11,812 4,727	71.42 28.58	11,789 4,699	71.50 28.50
**Household monthly income (baht)** ≥30001 ≥20001 - <30001 ≥10001 - <20001 <10001	300 418 999 14,589	1.84 2.56 6.13 89.47	281 409 974 14,875	1.70 2.47 5.89 89.94	325 390 1,005 14,768	1.97 2.37 6.10 89.57
Mean (SD)	3,741.27 (9,412.58)		3,549.46 (8,992.28)		3,624.93 (9,322.23)	
Median (Min - Max)	0 (0 - 345,000)		0 (0 - 325,000)		0 (0 - 325,000)	
**Residential area** Rural Urban	9,897 6,409	60.70 39.30	10,208 6,331	61.72 38.28	10,349 6,139	62.77 37.23
**Region** Bangkok Central North Northeast South	3,797 3,182 2,953 4,476 1,898	23.29 19.51 18.11 27.45 11.64	3,670 3,331 3,046 4,558 1,934	22.19 20.14 18.42 27.56 11.69	3,478 3,346 3,085 4,622 1,957	21.09 20.29 18.71 28.03 11.87

The bivariate analysis indicated that gender, age, marital status, smoking status, education, occupation, household monthly income, current liability, residential area and region were significantly (p-value <0.25) associated with HT in three consecutive years ( Table 2). 

**Table 2.** Frequency of hypertension (HT) in participants of the National Socioeconomic Survey for the years 2005–2007. This includes the odds ratio (OR) of having HT, with 95% confidence intervals (CI), for various characteristics of participants.

Characteristics	2005 (n = 16,306)				2006 (n = 16,539)				2007 (n = 16,488)			
	N	% with HT	OR (95% CI)	p-value	N	% with HT	OR (95% CI)	p-value	N	% with HT	OR (95% CI)	p-value
**Gender** Male Female	7,577 8,729	4.54 8.41	1 1.69 (1.29 - 2.20)	<0.001	7,673 8,866	4.46 7.77	1 1.81 (1.58 - 2.06)	<0.001	7,653 8,835	4.83 8.67	1 1.87 (1.64 - 2.12)	<0.001
**Age (years)** <35 ≥35 - <45 ≥ 45 - <55 ≥55 - <65 ≥65	5,937 3,570 2,933 1,854 2,012	0.91 3.75 8.73 12.73 19.78	1 4.25 (3.08 - 5.84) 10.42 (7.74 - 14.02) 15.89 (11.76 - 21.47) 26.84 (20.11 - 35.89)	<0.001	5,839 3,637 2,998 2,022 2,043	0.50 2.91 7.87 13.06 19.38	1 6.01 (3.98 - 9.09) 17.11 (11.61 - 25.24) 30.08 (20.43 - 44.31) 48.17 (32.91 - 70.51)	<0.001	5,635 3,556 3,061 2,110 2,126	0.53 2.70 8.40 13.98 21.54	1 5.18 (3.42 - 7.83) 17.12 (11.70 - 25.06) 30.37 (20.78 - 44.37) 51.30 (35.32 - 74.52)	<0.001
**Marriage status** Single Married Widowed/Separated	3,720 10,708 1,878	2.07 6.63 15.50	1 3.35 (2.65 - 4.26) 8.68 (6.70 - 11.23)	<0.001	3,718 10,886 1,935	1.37 6.55 13.80	1 5.04 (3.78 - 6.71) 11.51 (8.48 - 15.61)	<0.001	3,740 10,719 2,029	1.55 7.11 15.57	1 4.86 (3.71 - 6.36) 11.71 (8.80 - 5.58)	<0.001
**Smoking status** No Yes	13,124 3,182	3.78 18.29	1 3.15 (2.78 - 3.57)	<0.001	11,864 4,675	4.02 11.85	1 3.21 (2.83 - 3.65)	<0.001	13,433 3,055	3.85 20.26	1 3.60 (3.19 - 4.07)	<0.001
**Education** High (Upper primary school) Low (Primary school)	7,325 8,981	4.05 8.70	1 2.25 (1.96 - 2.59)	<0.001	7,592 8,947	3.60 8.47	1 2.48 (2.15 - 2.86)	<0.001	7,705 8,783	3.93 9.48	1 2.56 (2.34 - 2.93)	<0.001
**Status of work** No Yes	4,227 12,079	10.81 5.14	1 0.45 (0.40 – 0.51)	<0.001	4,348 12,191	10.58 4.68	1 0.42 (0.37 – 0.47)	<0.001	4,299 12,189	11.54 5.25	1 0.42 (0.38 – 0.48)	<0.001
**Occupation** Government officer Private business Personnel/employee Agriculture/labour	1,082 2,698 3,347 9,179	4.53 2.67 6.04 8.23	1 0.58 (0.40 - 0.84) 1.35 (0.98 - 1.86) 1.89 (1.41 - 2.54)	<0.001	3,267 1,308 3,653 8,311	3.70 2.22 6.87 7.58	1 0.59 (0.39 - 0.89) 1.92 (1.54 - 2.40) 2.13 (1.75 - 2.60)	<0.001	1,133 3,222 3,132 9,001	4.15 2.33 7.28 8.73	1 0.55 (0.38 - 0.79) 1.81 (1.32 - 2.50) 2.21 (1.64 - 2.98)	<0.001
**Household monthly** **Income (baht)** ≥30001 ≥20001 - <30001 ≥10001 - <20001 <10001	300 418 999 14,589	2.67 4.55 2.70 7.02	1 1.74 (0.75 - 4.02) 1.01 (0.46 - 2.26) 2.76 (1.36 - 5.58)	<0.001	281 409 974 14,875	4.27 3.42 2.46 6.59	1 0.79 (0.36 - 1.74) 0.57 (0.28 - 1.15) 1.58 (1.88 - 2.83)	<0.001	325 390 1,005 14,768	3.38 4.62 1.79 7.37	1 1.38 (0.64 - 2.97) 0.52 (0.24 - 1.11) 2.27 (1.24 - 4.16)	<0.001
**Current liabilities** No Yes	11,461 4,845	6.70 6.40	1 0.95 (0.83 - 1.09)	0.176	11,812 4,727	6.41 5.80	1 0.90 (0.78 - 0.94)	0.138	11,789 4,699	7.15 6.24	1 0.86 (0.75 - 0.99)	0.034
**Residential area** Rural Urban	9,897 6,409	6.28 7.11	1 1.14 (1.01 - 1.30)	0.038	10,208 6,331	5.81 6.92	1 1.21 (1.06 - 1.37)	0.004	10,349 6,139	6.19 8.06	1 1.33 (1.18 - 1.50)	<0.001
**Region** Bangkok Central North Northeast South	3,797 3,182 2,953 4,476 1,898	6.45 8.01 8.43 4.78 6.06	1 1.26 (1.05 - 1.52) 1.34 (1.11 - 1.60) 0.73 (0.60 - 0.88) 0.94 (0.74 - 1.18)	<0.001	3,670 3,331 3,046 4,558 1,934	6.76 7.39 7.68 3.97 6.31	1 1.10 (0.92 - 1.32) 1.15 (0.95 - 1.38) 0.57 (0.47 - 0.69) 0.93 (0.74 - 1.16)	<0.001	3,478 3,346 3,085 4,622 1,957	7.65 8.67 8.75 4.13 6.08	1 1.15 (0.96 - 1.36) 1.16 (0.97 - 1.38) 0.52 (0.43 - 0.63) 0.78 (0.63 - 0.98)	<0.001

**Figure 1.**
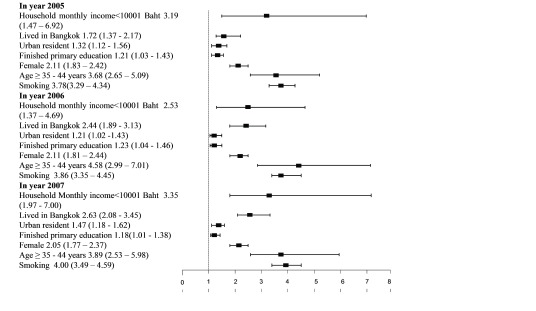
Adjusted odds ratios and 95% confidence intervals for characteristics associated with hypertension in participants of the National Socioeconomic Survey for the years 2005–2007.

The final model of the multiple logistic regression analysis after adjusting for covariates, which included gender, age, and smoking status, indicated that in 2005, 2006 and 2007, the odds of having HT were significantly higher among those who had household monthly income <10001 baht ^1*^ (AOR = 3.19; 95%CI: 1.47 to 6.92, AOR = 2.53; 95%CI: 1.37 to 4.69, and AOR= 3.35; 95%CI: 1.97 to 7.00, respectively), lived in Bangkok when compared with the Northeast region (AOR = 1.72; 95%CI: 1.37 to 2.17, AOR= 2.44; 95%CI: 1.89 to 3.13 and AOR=2.63; 95%CI: 2.08 to 3.45, respectively), lived in urban areas (AOR= 1.32; 95%CI: 1.12 to 1.56, AOR= 1.21; 95%CI: 1.02 to 1.43 and AOR= 1.47; 95%CI: 1.18 to 1.62, respectively), and only finished primary education (AOR =1.21; 95%CI: 1.03 to 1.43, AOR = 1.23; 95%CI: 1.04 to 1.46, and AOR= 1.18; 95%CI: 1.01 to 1.38 respectively). Other covariates that were statistically significant associated with HT were smoking (AOR= 3.78; 95%CI: 3.29 to 4.34, AOR= 3.86; 95%CI: 3.35 to 4.45 and AOR= 4.00; 95%CI: 3.49 to 4.59, respectively), aged 35 to 44 years old when compared with younger age groups (AOR= 3.68; 95%CI: 2.65 to 5.09, AOR= 4.58; 95%CI: 2.99 to 7.01 and AOR= 3.89; 95%CI: 2.53 to 5.98, respectively), and women (AOR= 2.11; 95%CI:1.83 to 2.42, AOR= 2.11; 95%CI:1.81 to 2.44, and AOR= 2.05; 95%CI: 1.77 to 2.37, respectively) ( Figure 1).

## Discussion

From the National Socioeconomic Survey data, we focused on examining the prevalence of HT and influences of SES on HT among Thai adults. HT is a common chronic disease and one of the most powerful contributors to CVD ^1, 2^. This study was conducted among the nationally representative samples with a large sample size; therefore, the results should be generalizable to represent the Thai population. In this study, SES factors, i.e. education, household monthly income, residential areas and region, were associated with HT even after adjusting for potential confounders.

Previous studies have revealed the association between the level of education and HT ^6– 9, 12, 18– 20, 23^, and they indicated that a low level of educational attainment was significantly associated with increased prevalence of HT. High education attainment is related to self-care and crucial to guarding against smoking. It can defeat related risk factors of HT by influencing a healthy lifestyle ^7, 18, 24^. Moreover, sound knowledge on health can affect individual behaviour in several ways, such as involvement in health promotional activities and accessing health services ^24^. Those with a higher education are provided with exponentially higher range and number of job opportunities and medical benefits packages, compared to those with lower levels of education. Another justification is that the higher education may promote the achievement of social gain, psychological support, and economic productivity by opening windows of opportunities. Thus, these performances can influence a person to socialize with peer groups that consequently promote god health behaviour, great self-esteem, and strong self-efficacy ^19, 20, 24^.

Previous studies have reported that low income is associated with HT ^19, 20^. These findings are consistent with our study that participants with the low income had a higher risk of HT than those with a high income. As previously stated, a key factor is an income that can highly influence the behaviour. It can satisfy mental health, food behaviour, and make one aware of accessing health care to promote sound health ^19^. Moreover, an income is essential to purchase better nutrition, high-quality education, healthy housing, and access to recreation. Previous studies also support these statements, saying that socioeconomic and psychosocial factors strongly affect individual health status ^20^. Therefore, having a good income can be a useful measure to examine the variables that transform the health of the population ^19^. A low income group have a higher tendency to develop HT and require treatment by changing lifestyles, such as weight loss, physical activity, and salt intake reduction ^20^. It is highly important in terms of public health to identify these individuals so as to set up measures to delay or prevent HT progression or development.

The results of this study also showed that the residential area and region were significantly associated with HT. HT prevalence was highest among those who lived in the North than other regions and the lowest was in the Northeast. People who were living in Northeastern and Southern regions of Thailand were less likely to have HT than those living in Bangkok metropolitan area. Similarly, a previous study indicated that HT prevalence was correlated with geographical region ^25^. For example, our study showed that there was the lowest prevalence of HT in the Northeast region, similar to the findings of a survey in Thai health working groups and health behaviour ^26^ and the National Health Examination Survey ^25^. In the Northeast, people were seldom aware that they had HT ^25^, which is similar to the findings of the Health and Welfare Survey of the NSO, Thailand ^5^. Residential areas where they lived could influence health behaviours in terms of lifestyle, social well-being and urbanization ^27^. The results of this study corresponded to previous findings in Mae Hong Son province in Thailand ^11^, Dehui City of Jilin province in China ^16^, and Northwest in Ethiopia ^16^, which reported a significantly higher tendency of HT in urban areas or cities rather than rural areas. Urbanization was associated with eating habit changes and obesity caused by reduced physical activity. Thai people have changed eating habits according to changing lifestyles between urban and rural residents ^28^. Such lifestyle and eating habit changes are conducive to a high prevalence of abdominal obesity in the urban population, eventually resulting in increased prevalence of HT. Similar to the previous study ^28^, residents in urban areas have a higher prevalence of being overweight or obese when compared with rural residents ^15, 18^. The differences in job opportunities and the quality of education in urban areas possibly impose an influence on the average socioeconomic accomplishment of its residents. The quality of the neighbourhood environment may be influenced by different levels of inequality regarding the distribution of social and economic resources across metropolitan areas. There have also been links of several aspects of the residential context to disparities in CVD risk and HT, including neighbourhood poverty and disadvantages, neighbourhood social cohesion, walking ability, availability of a healthy diet, and safety ^18^. The differences in environmental exposures are possibly linked to HT ^29^. In addition, neighbourhood-level SES could differently affect healthcare accessibility. Adverse neighbourhoods can increase levels of stress, and induce negative health behaviours, while failing to perform health promoting behaviours, possibly leading to HT. This study revealed that HT, which varied by each residential area, depended on the socio-environmental context at both metropolitan and neighbourhood levels ^29^.

In addition, the results from the multivariate analysis performed in this study indicated that covariate factors, such as gender, age and smoking, were strongly associated with HT. Women had a higher prevalence of HT than men over all three years, similar to the findings of a previous study in Thailand ^5^. In women, hormonal change after menopause has an effect on increasing blood pressure. The walls of a woman’s blood vessels can become less flexible when estrogen decreases, causing blood pressure (BP) to rise. The decline in estrogen levels can increase the risk for stroke and heart disease, especially due to high BP ^13^. Moreover, an older age had significantly higher odds of having HT than younger individuals. These findings are consistent with previous studies ^5, 6, 9– 12^. In older individuals, arteries harden, kidney function decreases, the body has a greater sensitivity to salt and other factors, and there are hormonal changes, such as menopause. Furthermore, aging is also associated with a decrease in heart rate, intravascular volume, stroke volume, renal blood flow, plasma renin activity and cardiac output, and an increase in left ventricular mass index and renal vascular resistance, resulting in higher BP when a progressive decline in the ability of the kidneys to excrete salt loads efficiently ^30^. In addition, elderly individuals are less likely to be physically active, which is also one of risk factor of HT. Thus, aging individuals are more likely to have an increasing risk of HT ^6, 17^. Additionally, this study indicated that HT was more prevalent among smokers. Indeed, smoking, in the form of cigarette or tobacco, can influence the deterioration of the overall health condition. Almost all physical systems, such as cardiovascular, cerebrovascular, respiratory, digestive, endocrine, urogenital and reproductive organs, can be affected by the harmful constituents of smoking. Smoking is an influential risk factor for developing cardiovascular-related diseases and morbidities, and discontinuation or cessation of smoking behavior can limit the process of initiating HT ^28^. Thus, smoking causes a series of actions, such as loss of endothelial functionality, arterial stiffness causation, and recurrence of inflammation within the body ^28^.

### Limitations

This study analysed nationally representative sample information. The findings indicated an increasing trend of HT and the association between the socioeconomic disparities and HT. It is noted that some variables, such as health behaviours, were not included in the study. However, these variables were found not strongly related to HT in previous studies when compared with demographics and smoking that were included in this study. Anyhow, we suggest that additional research focusing on biomolecular milieu, prenatal and early life exposures, historic SES conditions, health behaviours and their interplay in patients with HT may broaden the knowledge of associations among SES disparities and HT.

## Conclusions

This study supports previous findings indicating that being a women, middle aged to elderly, and smoking are strongly associated with HT. The study also reported a new conclusion that socioeconomic factors had a significant influences on HT. Populations with low educational attainment, low income, urban, and metropolitan residents (Bangkok) were vulnerable to HT. Above all, the interaction between SES and biology combined to accelerate bio-molecular characteristics that could differently impose influences on HT. These findings deliver important implications for future research and healthcare provision in relation to the prevention of HT. Health personnel and other relevant sectors should be aware of the significant roles of these SES disparities on HT in order to develop appropriate policies aimed at preventing HT.

## Data availability

Data used in this study were obtained from the NSO. Permission to use these data can be requested from the NSO. Researchers can request the NSS data by submitting an application form to the NSO Database Committee (the application form should be requested from the NSO Database Committee; services@nso.go.th). More details for submitting a request can be obtained from the NSO’s Statistical Information Service and Dissemination Group ( services@nso.go.th).

## Note

^1*^ The amount was equal to 248.35 US dollar, 263.68 US dollar and 289.35 US dollar, in year 2005, 2006 and 2007, respectively. These conversions used the official exchange rates obtained from the Bank of Thailand.
