# Supplementary material for: Socioeconomic disparities in income, education and geographic location for hypertension among Thai adults: Results from the National Socioeconomic Survey
Source: F1000Res. 2017 Oct 13;6:1836. [Version 1] doi: 10.12688/f1000research.12709.1 (PMC6080422; doi:10.12688/f1000research.12709.1)
Supplement: Peer review history

# Peer review: Referee response for version 1

**DOI:** 10.5256/f1000research.13767.r31475

**Published:** 2018-04-09

**Authors:** Abha Shrestha, Anmol Shrestha

**Affiliations:** 1 Department of Community Programs, Dhulikhel Hospital, Kathmandu University Hospital, Dhulikhel, Nepal; 2 Department of General Practice, Dhulikhel Hospital, Kathmandu University Hospital, Dhulikhel, Nepal

The authors have presented the growing burden of hypertension in Thailand and its association with the socioeconomic status. The paper is relevant to the current scenario of increasing burden of Noncommunicable diseases. 

1. I have a suggestion, please add the reference: Kearney et al. (2005) ^1^ to the reference number 4.
2. Since, this study is about hypertension, it would be nice to explain more on the diagnosis criteria used for hypertension. 
3. The result section of the abstract is not very clear.

We have read this submission. We believe that we have an appropriate level of expertise to confirm that it is of an acceptable scientific standard.

## References

[1] KearneyPWheltonMReynoldsKMuntnerPWheltonPHeJ: Global burden of hypertension: analysis of worldwide data. *The Lancet*.2005;365(9455) : 10.1016/S0140-6736(05)17741-1 217-223 10.1016/S0140-6736(05)17741-1 15652604

# Peer review: Referee response for version 1

**DOI:** 10.5256/f1000research.13767.r30222

**Published:** 2018-02-06

**Authors:** Matthew Kelly

**Affiliations:** 1 National Centre for Epidemiology and Population Health, Australian National University, Canberra, ACT, Australia

The authors present a straightforward account of an important and growing health burden in Thailand, hypertension. Given the increasing prevalence of this condition and the associated increased risk for other chronic diseases this paper is timely and relevant. The identification of socio-economic drivers is particularly important in a country such as Thailand which has experienced rapid economic growth and change in the recent past.

 I have a few comments as follows:

1. Why are the authors using 2005 to 2007 data? These data are now more than 10 years old and given the rapid rise in HT prevalence described by the authors the most up to date data are important.
2. The results section of the abstract needs to be rewritten. It describes associations between for example income and education and HT but does not describe the direction of the association. That is, high income or low income that associates with hypertension.
3. In the Methods HT is measured by self-report of a physician diagnosis. Has any validation analysis been done to assess the accuracy of this type of self report? Also this leaves the strong possibility of undiagnosed HT being prevalent in the community. This cannot be measured using this study design but it should be mentioned in the limitation section of the discussion. 
4. Also in the Methods the authors describe a sample of approximately 16,000 people in each study year. What proportion is this of the total study population after exclusions were made. 
5. Was there any non-response or refusal to participate rate that should be reported?
6. In the bivariate analysis variables were included where their association with hypertension was significant at p<0.25. Why was this value chosen?
7. The Discussion section of this paper needs some work. 
  a. The authors discuss education and income as indicators of socio-economic status and observe that low education and low income are associated with HT risk. This is the case in contemporary Thailand. It may be worth discussing here though that this is a modern phenomenon and the result of health and epidemiological transitions. Previously in most low and middle income countries chronic disease risk including obesity and HT was concentrated in the higher socio-economic groups. 
  b. There is a little bit of a contradiction between the education/ income – HT association and the geographic association which needs some more consideration. Incomes are generally higher in urban areas which should according to the SES hypothesis mean that they have the lowest levels of HT. However in this study, the Northeast, the poorest area of the country has the lower prevalence. As the authors suggest this could be because in Isan the undiagnosed HT may be higher. But there also may be different directions of association between income and HT depending where you live. I am not sure there will be a clear answer to this but it deserves some discussion in the paper.

I have read this submission. I believe that I have an appropriate level of expertise to confirm that it is of an acceptable scientific standard.

# Peer review: Referee response for version 1

**DOI:** 10.5256/f1000research.13767.r27016

**Published:** 2017-11-10

**Authors:** Limin Wang, Yichong Li

**Affiliations:** 1 National Center for Chronic and Non-communicable Disease Control and Prevention, Chinese Center for Disease Control and Prevention, Beijing, China; 2 Peking University Health Science Center, Beijing, China

Comments:

1. Language in abstract is unreadable, especially in the results part.
2. Please provide the sample size for each stage of sampling, if equal sample size strategy was used.
3. What is the ultimate sampling unit? If household, were all individuals in the selected household included in the survey?
4. Did the analysis take into account the multistage sampling design? If not, the present estimations were not reliable, as sampling error might get probably underestimated.
5. The results for every study year are much similar, and the SES effects on HT should not change during short period. So, what is the point of annually independent analysis?

We have read this submission. We believe that we have an appropriate level of expertise to confirm that it is of an acceptable scientific standard, however we have significant reservations, as outlined above.
